# Breaking Boundaries: Immunotherapy for Myeloid Malignancies

**DOI:** 10.3390/cancers16162780

**Published:** 2024-08-06

**Authors:** Tatyana Gavrilova, Eduard Schulz, Alain Mina

**Affiliations:** 1 National Heart, Lung, and Blood Institute, National Institutes of Health, Bethesda, MD 20892, USA; 2 Immune Deficiency—Cellular Therapy Program, Center for Cancer Research, National Cancer Institute, National Institutes of Health, Bethesda, MD 20892, USA; eduard.schulz@nih.gov (E.S.); alain.mina@nih.gov (A.M.); 3 NIH Myeloid Malignancies Program, National Institutes of Health, Bethesda, MD 20892, USA

**Keywords:** myeloid malignancies, immunotherapy, leukemia, myelodysplastic syndrome

## Abstract

**Simple Summary:**

Patients with myeloid malignancies (blood cancers such as leukemia and myelodysplastic syndromes) that have returned or worsened despite chemotherapy or a stem cell transplant face limited treatment choices. Immunotherapy holds promise in deploying the body’s own immune system to target and destroy cancer cells that are otherwise evading immune detection. This review article summarizes the most up to date information on immunotherapy options for patients with myeloid malignancies as well as discusses the challenges faced in this evolving field.

**Abstract:**

Immunotherapy has revolutionized the treatment of myeloid oncologic diseases, particularly for patients resistant to chemotherapy or ineligible for allogeneic stem cell transplantation due to age or fitness constraints. As our understanding of the immunopathogenesis of myeloid malignancies expands, so too do the treatment options available to patients. Immunotherapy in myeloid malignancies, however, faces numerous challenges due to the dynamic nature of the disease, immune dysregulation, and the development of immune evasion mechanisms. This review outlines the progress made in the field of immunotherapy for myeloid malignancies, addresses its challenges, and provides insights into future directions in the field.

## 1. Introduction

Immunotherapy has shown great promise in treating hematologic malignancies, particularly in lymphoid malignancies. However, its application in myeloid malignancies has not yielded the same success in identifying targeted therapies capable of inducing lasting remissions in both treatment-naïve and heavily pretreated patients. Allogeneic hematopoietic stem cell transplantation (allo-HSCT) remains the only potentially curative treatment option for many myeloid malignancies, particularly myelodysplastic neoplasms (MDS) and non-favorable risk acute myeloid leukemia (AML) [1,2,3]. However, allo-HSCT has significant limitations, including donor availability, relapse risk, patient fitness, and the ability to tolerate high-intensity chemotherapy, as well as short- and long-term toxicity and morbidity. 

MDS and AML are characterized by significant genetic and phenotypic heterogeneity. MDS is marked by ineffective hematopoiesis, dysplasia, persistent cytopenias, and the risk of leukemic transformation. It is stratified into different prognostic risk categories using tools such as the International Prognostic Scoring System (IPSS) [4], revised IPSS (IPSS-R) [5], and molecular IPSS (IPSS-M) [6,7]. AML is characterized by the accumulation of undifferentiated myeloid precursor cells in the bone marrow disrupting normal hematopoiesis. From a biological and clinical behavior perspective, the boundary between MDS and AML as distinct entities can be blurred, as these diseases may be perceived as existing along a continuum. In order to address their similarities and facilitate access to clinical trials, the International Consensus Classification (ICC) introduced the new entity MDS/AML, which is defined by 10–19% blasts in the bone marrow or peripheral blood [8]. In the future, trials for novel drugs developed for AML may also investigate their efficacy in MDS/AML.

**Figure 1:**
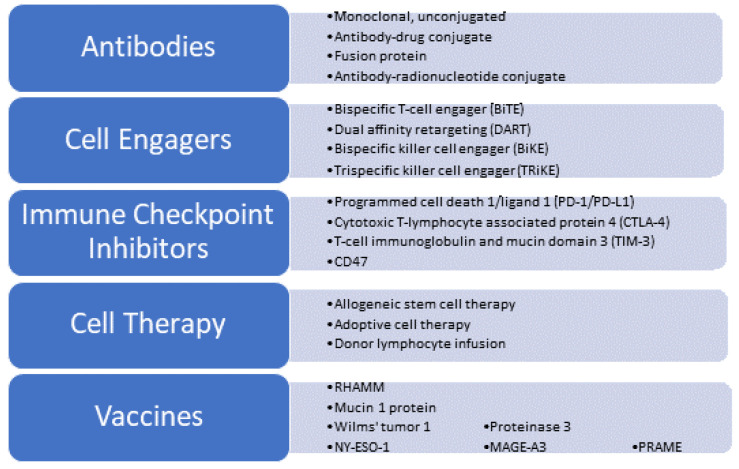
Immunotherapy for myeloid malignancies.

Hypomethylating agents (HMAs) such as azacitidine (AZA) and decitabine (DEC) are the recommended frontline therapy for transplant-ineligible higher-risk MDS. Outcomes after failure of upfront HMA in high-risk MDS are dismal, and phase II studies investigating treatments for patients with relapsed/refractory (R/R) MDS have not produced a median overall survival (OS) beyond 1 year [9,10]. Combining HMA with venetoclax (VEN) has emerged as the standard of care for patients with newly diagnosed AML (ND-AML) who cannot tolerate intensive induction chemotherapy due to comorbidities [11]. However, this treatment is not considered to be definitive, and consolidation therapies are often necessary to achieve a cure. This highlights the need to investigate novel approaches for patients who have not responded to standard therapies. Immunotherapy can potentially offer a more targeted approach with less systemic toxicity. This review will examine the current landscape of immunotherapy treatments for MDS and AML, as well as their limitations (Figure 1).

## 2. Immunotherapy for Myeloid Malignancies: Finding the Right Target

Immune cells require the recognition of a target to operate effectively. In AML, recognized antigens include leukemia-specific neoantigens, leukemia-associated antigens, and lineage-restricted antigens [12]. Leukemia-specific neoantigens arise from leukemogenic mutations, gene fusions, and cancer-specific splicing variants that are expressed extracellularly solely by leukemic clones and presented as membrane proteins or in conjunction with an HLA molecule [13,14]. Leukemia-associated antigens are not lineage-specific, and while they are found in non-leukemic cells, they are overexpressed in AML cells [15]. Since these antigens are found on non-hematopoietic tissues, targeting them can lead to on-target off-tumor toxicities [16]. Examples of such antigens include WT1 and PRAME [17]. Lineage-restricted antigens are limited to the myeloid lineage, with examples including CD33 and CD123 [18]. Currently, most immunotherapy agents developed for myeloid malignancies target lineage-restricted antigens.

The tumor microenvironment (TME) is a complex milieu of cells that supports the survival of cancer cells [19]. Comprising of stromal cells, immune cells, and vasculature, the TME promotes the upregulation of inhibitory ligands and the production of immunosuppressive factors, which allow the proliferation of tumor cells. An example of such immunosuppressive mechanisms involves myeloid-derived suppressor cells (MDSCs) which have been shown to inhibit T cells [20]. Additionally, a subset of macrophages known as M2 macrophages produce soluble factors such as transforming growth factor beta (TGFβ) and vascular endothelial growth factor (VEGF), which can remodel the tumor matrix and further inhibit T-cell function [21].

Another strategy for tumor escape within the TME involves the evasion of immune checkpoint inhibition. This occurs when cancer cells express ligands that are typically expressed by the immune system in order to maintain homeostatic immune tolerance but, in the case of neoplastic disease, promote tumor survival [22]. One such potent immune mechanism involves inducing co-inhibitory receptors such as programmed cell death protein 1 (PD-1) and programmed cell death ligand 1 (PD-L1), cytotoxic T-lymphocyte associated protein 4 (CTLA-4), T cell immunoglobulin and mucin domain 3 (TIM-3), T cell immuno-receptor with Ig and ITIM domains (TIGIT), and lymphocyte activating-3 (LAG-3). Blocking these co-inhibitory molecules has emerged as a strategy to enhance antitumor immunity [23].

### 2.1. Antibodies

#### 2.1.1. CD33

CD33 is a differentiation protein expressed on over 80% of AML blasts and also expressed on normal myeloid precursors [24]. A high CD33 expression has been associated with worse clinical outcomes in AML [25]. Lintuzumab (HuM195) is an early highly specific anti-CD33 humanized monoclonal antibody that induces antibody-dependent cell-mediated cytotoxicity. However, it demonstrated limited improvement in the survival and response rates of patients with relapsed/refractory (R/R) AML [26]. Gemtuzumab ozogamicin (GO) is another CD33-directed antibody, but unlike lintuzumab, it is conjugated to the cytotoxic calicheamicin. An initial phase 3 study of patients with ND-AML receiving daunorubicin and cytarabine (DA) with or without GO at a dose of 6 mg/m^2^ on day 4 showed a trend toward superior survival in the DA-only group. Relapse-free survival (RFS) was better in the DA+GO group among patients with favorable cytogenetics (hazard ratio = 0.49; *p* = 0.043). However, the results of this trial failed to demonstrate improvement in the complete response (CR) rate, RFS, or overall survival (OS) when GO was added to either induction or post-consolidation therapy. In addition, the rate of fatal induction toxicity was significantly higher in the DA+GO group compared to the DA-only group (5.5% versus 1.4%) [27]. GO was then voluntarily removed from the market by its manufacturer. However, AML15 and AML16, two subsequent randomized trials that used GO at a dose of 3 mg/m^2^ in patients with ND-AML, demonstrated a survival benefit for favorable-risk disease and a trend toward benefit in the intermediate-risk group [28,29]. Importantly, the addition of GO at this lower dose was well tolerated, with no significant increase in toxicity. AML16 demonstrated a better RFS for the GO arm (28% versus 23% at 2 yrs; *p* = 0.03). A meta-analysis of five randomized GO studies confirmed survival benefits at 5 years for favorable-risk ND-AML when GO was added to induction [30].

The UK NCRI AML18 trial demonstrated that fractionated GO administration to older adults (median age of 68 years) diagnosed with AML/high-risk MDS resulted in a greater reduction in measurable residual disease (MRD) and improved survival, particularly in those with non-adverse cytogenetics. The study randomized patients to single-dose GO 3 mg/m^2^ (dose not capped) on day 1 (GO1) versus two doses of GO 3 mg/m^2^ (maximum dose 5 mg) on days 1 and 4 (GO2) of induction. The GO2 arm showed a higher rate of CR compared to the GO1 arm, particularly among those with MRD < 0.1% (50% versus 41%; odds ratio = 0.72; *p* = 0.027) [31]. Of note, patients with *IDH1/2* mutations experienced the greatest reduction in MRD. In a sensitivity analysis that excluded patients with adverse cytogenetics or *TP53* mutations, the 5-year OS was 33% for the GO2 group and 26% for the GO1 group (HR = 0.83; *p* = 0.045). Adverse events occurred with comparable frequencies and severities between the two arms. However, the platelet recovery times were longer in patients with secondary AML in the GO2 arm as compared to GO1 (median days 32 for GO2 versus 30 days for GO1; *p* = 0.027). Otherwise, there were no significant differences between the two groups regarding the time to neutrophil and platelet recovery [29].

The ALFA-0701 trial investigated the impact of adding fractionated doses of GO to standard DA chemotherapy for the treatment of ND-AML in patients aged 50–70 years. The primary endpoint of EFS at 2 years was significantly different between the control and GO groups, with rates of 17.1% and 40.8%, respectively (HR = 0.58; *p* = 0.0003). There were also statistically significant differences between the control and GO groups in OS (41.9% versus 53.2%, *p* = 0·0368) and RFS (22.7% versus 50.3%, *p* = 0.0003). Persistent thrombocytopenia was more common in the GO group (16%) compared to the control group (3%), without an associated increase in the risk of death [31]. In 2017, the FDA re-approved single-agent GO for the treatment of R/R AML [32]. Presently, it is approved for use in combination with induction chemotherapy in medically fit, previously untreated patients with CD33-positive AML [33]. Additionally, its use is supported in patients with high-risk APL (WBCs ≥ 10 × 10^9^/L) in addition to arsenic trioxide (ATO) and all-trans retinoic acid (ATRA) [34].

Lintuzumab-Ac225 (Actimab-A) is an antibody-radionuclide conjugate (ARC) that combines the alpha emitter actinium-225 with the anti-CD33 monoclonal antibody lintuzumab. A phase I study reported outcomes of lintuzumab-Ac225 administration in high-risk patients with CD33+ R/R AML in combination with cladribine, cytarabine, filgrastim, and mitoxantrone (CLAG-M). Among the 19 patients enrolled, 16 had adverse-risk disease according to the European Leukemia Network (ELN) criteria, 75% of whom (n = 12) harbored *TP53* mutations. In high-risk patients, 42% (n = 8) achieved a composite CR (CRc = CR plus CR with incomplete count recovery) while 16% (n = 3) achieved a morphologic leukemia-free state (MLFS), resulting in an objective response rate (ORR) of 58%. The median OS was 7.3 months in those with *TP53* mutations [35]. An ongoing open-label phase 1/2 trial is investigating the combination of lintuzumab-Ac225 with VEN in CD33+ R/R AML (NCT03867682). The long-term effects of alpha radionuclide therapy targeting the hematopoietic system are still unclear.

#### 2.1.2. CD123

CD123, the α chain of the interleukin 3 receptor, is overexpressed in a number of hematologic neoplasms, including AML, blastic plasmacytoid dendritic cell neoplasm (BPDCN), and acute lymphoblastic leukemia [36]. Tagraxofusp-erzs (TAG) is a CD123-directed fusion protein consisting of recombinant IL3 fused to a truncated diphtheria toxin (DT) payload. Following endocytosis, the DT catalytic domain is released from endosomes, leading to cell death through the inhibition of protein synthesis [37]. TAG was FDA approved as a single agent in 2018 for BPDCN, an aggressive hematologic malignancy characterized by cutaneous and/or systemic involvement and CD123 positivity [38]. In vitro studies demonstrated cytotoxicity against CD123-positive AML cells, which prompted the investigation of this drug in AML [39].

TAG-exposed AML cells were found to be sensitized to the BCL2 inhibitor VEN. Lane et al. reported the results of a phase 1b trial combining TAG with AZA or AZA and VEN in patients with AML, MDS, or BPDCN. The combination appeared to have activity primarily for ND disease. However, no patients with R/R AML who received tagraxofusp + AZA ± VEN (n = 17) achieved remission. Eight of nine patients with previously untreated AML (seven of nine ELN adverse risk) who received TAG-AZA-VEN achieved a best response of either CR (n = 5) or CRi (n = 3). The majority of these patients were classified as high risk, exhibiting features such as a *TP53* mutation, adverse karyotype, and secondary AML. Among the four patients with previously untreated MDS/AML, three received TAG-AZA and responded, achieving CR (n = 2) or marrow CR (n = 1). All three had *TP53* mutations [40]. A subsequent expansion cohort further validated the efficacy of this combination in ND-AML, particularly in high-risk patients. In this cohort, 26 patients with AML were treated, all of whom were classified as having an adverse risk according to the ELN 2022 criteria. The median follow-up duration was 10.7 months, median OS was 14 months, and PFS was 8.5 months. Eighteen of twenty-six patients (69%) achieved a best response of CR (n = 10; 39%), CRi (n = 5; 19%), or morphologic leukemia-free state (MLFS) (n = 3; 12%) [35]. For the 18 patients achieving CR, CRi, or MLFS, the median duration of response was 12.4 months. MRD was assessed by flow cytometry in 17 patients who achieved CR, CRi, or MLFS, and among these, 12 out of 17 (71%) tested MRD negative (<0.1%). Subsequently, 13 out of 26 patients (50%) proceeded to allo-HSCT with a median OS of 18.2 months and a median PFS of 13.3 months [41]. The most significant adverse event associated with tagraxofusp therapy is capillary leak syndrome. Careful monitoring and the early implementation of supportive care measures are required in order to prevent this potentially lethal toxicity [38,42].

Stephansky et al. reported a mechanism of resistance exhibited by AML and BPDCN cells after treatment with tagraxofusp that stems from DNA methylation-driven downregulation of diphthamide genes that code for the protein targets of the DT component of TAG. This resistance is reversed by treatment with the HMA azacitidine, which restores diphthamide biosynthesis 1 (DPH1) expression [40,41].

Pivekimab sunirine (PVEK/IMGN632) is a first-in-class ADC of a humanized anti-CD123 antibody G4723A tethered to a DNA-alkylating payload of the idolinobenzodiazepine pseudodimer (IGN) class of compounds [43]. Preclinical data from AML xenograft models have demonstrated synergy in IMGN632 combinations with AZA and VEN, supporting the exploration of these combinations in patients with AML [44]. This led to the investigation of the AZA/VEN/PVEK triplet in a phase 1b/2 clinical trial of high-risk patients with R/R AML. In this trial, the combination demonstrated anti-leukemic activity across multiple high-risk genetic subsets of R/R AML. Out of 61 evaluable patients with R/R AML, the ORR was 51% with a composite complete remission (cCR = CR + CR with partial hematologic recovery [CRh] + CR with incomplete platelet recovery [CRp] + CR with incomplete blood recovery [CRi]) of 31%. VEN-naive patients had an ORR and cCR of 62% and 47%, respectively, compared to 37% and 11%, respectively, in patients with prior VEN exposure. Notably, responses were observed in 9 out of 11 patients with AML harboring a FMS-like tyrosine kinase-3 (*FLT3*) receptor internal tandem duplication (ITD), with an ORR of 82% and a cCR of 64%. The most common treatment-emergent adverse event (TEAE) was grade 3+ febrile neutropenia, observed in 30% of patients, while infusion-related reactions were seen in 21%. Pneumonia was reported in 16% of patients [45].

### 2.2. Cell Engagers: BiTEs and DARTs

Immune cell engagers are engineered antibodies designed to simultaneously engage a tumor-associated antigen and an immune effector cell. This interaction allows for tumor cell lysis and cytokine release to take place, killing the tumor cell [46]. A bispecific T-cell engager (BiTE) is a hybrid molecule consisting of two antigen-binding sites derived from two antibodies. These binding sites can simultaneously bind two different antigens, with one site binding the CD3 cell-surface protein of T cells and the other targeting the cancer cell [47]. Blinatumomab, a monovalent CD3 and CD19 BiTE, was the first T-cell engager to receive FDA approval in 2014 for the treatment of acute lymphoblastic leukemia [48]. Despite its effectiveness, blinatumomab has a significant drawback due to its small size and lack of an Fc region, resulting in a short half-life requiring continuous infusion. Bispecific antibodies that feature Fc regions interact with immune effector cells expressing the corresponding Fc receptors and this rescues the antibody from cellular degradation [49]. Effector cells such as natural killer (NK) cells, activated macrophages, and dendritic cells, also release cytotoxic cytokines, a process known as antibody-dependent cellular toxicity (ADCC), further potentiating the effects of the antibody. Additionally, the Fc region binding to its receptor also activates antibody-dependent cellular phagocytosis (ADCP), a process in which antibody-opsonized target cells are phagocytosed by Fc receptor-activated macrophages [50].

While no additional single-chain BiTEs have yet reached the market after the approval of blinatumomab, several IgG antibody-based T-cell engagers have been approved for the treatment of R/R B-cell lymphomas [51] and R/R multiple myelomas [46].

AMG330, a human BiTE single-chain CD3ԐXCD33 antibody, has shown promise in enhancing T-cell activation and PD-L1 expression in long-term cultures of primary AML blasts [52]. However, one major mechanism of resistance to bispecific antibodies like AMG330 is immune evasion through the upregulation of PD-1 on activated T-cells. This observation provided the rationale for combining AMG330 with pembrolizumab in a clinical trial. The study, however, was terminated early due to insufficient evidence of efficacy (NCT 04478695).

AMV564 is a novel bivalent, bispecific CD3XCD33 T-cell engager. Its larger molecular weight, as compared to monovalent BiTEs, leads to reduced renal clearance and a longer half-life of 2–3 days, a significant improvement over blinatumomab’s 2.1 h [53]. A phase 1 trial of AMV564 in 36 patients with R/R AML demonstrated notable objective responses, including 1 CR, 1 CRi, 1 partial response (PR), and 3 patients experiencing hematologic improvement in neutrophil counts. The median duration of treatment was 20 days. Among participants, 24 patients (67%) had secondary AML and/or adverse cytogenetics, with 9 of the patients (25%) carrying a *TP53* mutation. No dose-limiting toxicities were reported, and no grade 3 or higher CRS was observed. The most common grade ≥3 treatment-emergent AE was anemia reported in 11% of patients [54].

Vibecotamab is a humanized bispecific antibody that monovalently binds tumor antigen CD123 to CD3, thereby recruiting cytotoxic T cells for the eradication of CD123+ tumor cells. In a phase 1 study of vibecotamab monotherapy in patients with R/R AML, a modest efficacy was observed, with 9.0% (10 of 111) of efficacy-evaluable patients achieving a response of MLFS or better. The occurrence of CRS was found to be dose-dependent. Notably, responses were correlated with lower baseline blast counts and a low PD-1 expression on both CD4+ and CD8+ T cells, and were predominantly observed in patients receiving a target dose of 0.75 μg/kg or higher [55]. A phase 2 study is currently underway (NCT05285813).

A dual-affinity retargeting protein (DART) represents a further modification to improve the limitations of a BiTE. The binding efficiency and conformational flexibility of a BiTE are constrained by the linker sequence between the two antigen-binding sites. In contrast, the configuration of a DART lacks the intervening linker sequence and instead features two cysteine residues at the C-terminus of each chain forming a disulfide bridge [51].

Flotetuzumab, a CD3εXCD123 DART, was evaluated in a phase 1/2 study involving heavily pretreated patients with R/R AML (n = 88). At the recommended phase 2 dose (RP2D) of 500 ng/kg per day, the CR or CR with partial hematologic recovery (CRh) rate was 30%, with a median OS of 10.2 months in patients who achieved CR/CRh. Interestingly, 47% of patients with *TP53* mutations (n = 15) achieved blast reduction to <5%. In the dose expansion group of patients treated at the RP2D (n = 50), infusion-related reactions (IRRs) and CRS were observed in the majority of subjects (96%), with four of them (8%) experiencing grade 3 or higher adverse events [56].

MGD024 is a second-generation CD3XCD123 DART, bearing an IgG1 Fc domain that prolongs its circulating half-life and allows for intermittent delivery designed to reduce the risk of CRS. Currently, a phase 1 study of MGD024 in patients with R/R myeloid malignancies is underway (NCT05362773).

Incorporating additional binding domains enhances an immune engager’s ability to bind both tumor and immune cells. MP0533 is a CD3-engaging protein that simultaneously targets CD33, CD123, and CD70 and has been shown to induce the T-cell-mediated eradication of tumors in AML xenograft mouse models [57]. There is currently an ongoing phase 1 study of MP0533 in patients with R/R AML and MDS (NCT05673057). Multi-specific NK cell engagers known as bispeficic and trispecific killer cell engagers (BiKEs and TRiKEs, respectively) are also in early phase trials. These include CD33/CD16/NKG2D (NCT04789655) for AML and CD123/CD16/NKp46 (NCT05086315) for AML and MDS [58].

### 2.3. Immune Checkpoint Inhibitors 

Increased expressions of programmed cell death protein 1 (PD-1) on T cells and programmed death ligand 1 (PD-L1) on blasts have been demonstrated in patients diagnosed with AML and MDS, particularly in those with R/R disease, correlating with an unfavorable prognosis [59]. PD-L1 expression has been shown to be elevated in patients with AML and has been associated with a poor overall survival [60]. An increased expression of PD-1 corresponds to T-cell exhaustion in AML [61]. The upregulation of PD-L1 is found on CD34+ hematopoietic stem cells (HSPCs) and CD71+ erythroid progenitors from patients with MDS [62].

The ICIs investigated in myeloid malignancies include those targeting T-cell checkpoints PD-1 [63], PD-L1 [64], and CTLA-4 [63], as well as the macrophage checkpoint CD47 [65]. Additionally, inhibitors of T-Cell Immunoglobin and Mucin Domain 3 (TIM-3), a co-inhibitory receptor expressed on IFN-y-producing T cells, macrophages, and dendritic cells have been explored [66].

The tumor mutation burden (TMB) is defined as the total number of nonsynonymous mutations per sequenced coding area of a tumor genome [67]. These mutations may be translated into neoantigens that are then presented on the cell surface and recognized by immune cells, thus making the tumor more immunogenic [68]. The hypothesis is, therefore, that a high TMB signifies a potential increase in neoantigen production that can, therefore, serve as a surrogate marker for response to ICI therapy [69]. TMB, however, has its limitations. Firstly, proteins generated by fusion or post-translational modifications are not accounted for with TMB. Additionally, every mutation may not result in a neoantigen, as only a minority of peptides are properly processed and presented in the context of an MHC complex, and fewer still are immunogenic enough to be recognized by T cells [62]. Therefore, a high TMB does not truly represent a high neoantigen burden, nor is every neoantigen a high-quality immunogen.

AML itself has a low TMB [61], and clinical trials of ICIs in AML have shown limited efficacy [60]. Checkpoint inhibitors have not proven to have significant efficacy in MDS either, particularly as monotherapy, and although ICIs have shown some synergistic effects when combined with hypomethylating agents, potentially reversing epigenetic modifications that otherwise hinder immune therapy [64], clinical trials have not demonstrated benefits. A randomized phase 2 trial of azacitidine with or without durvalumab, an antibody that binds PD-L1 and blocks PD-1/PD-L1 interaction, as a first-line therapy for higher-risk MDS did not show significant improvement in clinical outcomes over azacitidine alone, while demonstrating higher rates of toxicities in the ICI combination arm [70]. A phase II study of nivolumab or ipilimumab with or without azacitidine for patients with R/R MDS demonstrated the lowest ORR for either single-agent ICI and, consequently, the nivolumab arm closed due to a lack of clinical efficacy [71].

#### 2.3.1. CD47

CD47 is a widely expressed transmembrane protein that serves as the ligand for SIRPα, an inhibitory immunoreceptor expressed on macrophages and dendritic cells. Upon the binding of CD47, SIRPα is activated to transduce an anti-phagocytic signal [72]. CD47 expression is higher in leukemia stem cells (LSCs) compared to normal progenitor counterparts, allowing LSCs to evade elimination by phagocytosis. Antibodies targeting CD47 have been shown to promote the phagocytosis of LSCs while sparing normal hematopoietic stem cells [65]. MDS cells are also known to overexpress CD47, and its overexpression correlates with HR-MDS [73].

Magrolimab is a first-in-class anti-CD47 monoclonal antibody that has been investigated for the treatment of myeloid neoplasms. Early phase trials of this agent identified its potential role in the treatment of high-risk MDS and AML patients. A phase 1b trial of magrolimab in combination with AZA in high-risk MDS patients (n = 95) was notable for being the only completed study that included a high-risk patient cohort. This cohort comprised of 26% patients with *TP53* mutations, 62% with a poor/very poor cytogenetic risk according to the IPSS-R criteria, 67% with excess blasts, and 22% with therapy-related disease. The CR rate observed was 33%, with a 42% rate of flow cytometric MRD negativity and a median CR duration of 11 months. Magrolimab was also noted to have activity in *TP53*-mutated MDS cases (n = 25), with a median CR duration of 7.6 months, median PFS of 11.0 months, and median OS of 18.7 months for patients who underwent allo-HSCT (n = 4), compared to 12.1 months for those who did not undergo allo-HSCT [74]. In a phase 1/2 study evaluating the triplet combination of magrolimab/AZA/VEN for ND and R/R AML patients, the ND cohort (n = 41) exhibited a high prevalence of adverse risk disease (92%) and *TP53* mutations (66%). Among TP53-mutated AML cases, a notable 63% achieved CR or CRi, with a corresponding 1-year OS of 53%. The outcomes were also favorable for *TP53* wild-type disease, with CR/CRi rates of 83% and a corresponding 1-year OS of 83%. Notably, the triplet appeared to have limited activity in the R/R patients with prior VEN exposure (n = 17). For these patients, the CRi rate was 12%, with a median OS of 3.1 months. In contrast, the VEN-naïve R/R patients (n = 12) had a higher CR/CRi rate of 58% with a median OS of 7.4 months [69].

Sallman et al. reported the results of a phase 1b study that included patients with AML and MDS who received magrolimab. All 13 untreated MDS patients had an objective response: 7 patients (54%) achieved a CR, 5 (39%) achieved a marrow CR (of which 3 saw hematologic improvement), and 1 (7%) experienced hematologic improvement alone [75]. Unfortunately, despite promising results in earlier trials, the clinical efficacy of this agent was limited in phase 3 trials [76]. The ENHANCE-3 trial, which evaluated the first-line combination of magrolimab and azacitidine in patients with HR-MDS was discontinued in 2023 due to futility. There are currently no reported ongoing active efforts looking at magrolimab, as its phase 1/2 successes did not translate into phase 3 efficacy.

A number of different hypotheses have been proposed to explain the limited efficacy seen with CD47-targeting strategies. One limitation could be an increased expression of CD47 on tumor cell surfaces or an upregulation of alternative phagocytic mechanisms [21]. Predicting responders to CD47 targeting is also a limiting factor that might make patient selection difficult until a reliable biomarker is validated [77]. Perhaps the complexity of the tumor microenvironment and the heterogeneity of myeloid malignancies present the most difficult challenges for investigators [78]. Despite these obstacles, efforts to optimize the therapeutic potential of the CD47–SIRPα axis continue, given the promising signals of efficacy in hematological and solid tumors. One effort combines anti-CD47 antibodies with CAR-T cells engineered to express the CD47E variant CD47 (Q31P) (47E), which still engages SIRPα providing the “don’t eat me” signal. These CAR-T cells express the 47E variant, making them resistant to macrophage-mediated clearance [79]. The addition of anti-CD47 antibodies allows persistent macrophage recruitment to the tumor microenvironment and could be applicable across different tumor types. Other products currently in production include SIRPα-αCD123 antibodies and SIRPα-related fusion proteins [80], but none have yet made their way into clinical practice.

#### 2.3.2. T-Cell Immunoglobin and Mucin Domain 3 (TIM-3) Inhibitor

TIM-3, a negative immune regulator, is expressed on monocytes, dendritic cells, and effector T cells. Its interaction with its soluble ligand, galectin-9, which is secreted by tumor cells, leads to the suppression of T-cell activation [81]. TIM-3 is preferentially expressed on LSCs and myeloid blasts as compared to normal hematopoietic stem cells [82]. This immuno-myeloid regulator may have a potential role in activating T cells in the treatment of AML and MDS when combined with a hypomethylating agent.

Sabatolimab (MBG453) is a high-affinity humanized IgG4 antibody targeting the TIM-3 receptor on both immune and leukemic cells, as well as soluble TIM-3 [83]. Borate et al. reported the results of a phase 1b study of sabatolimab in combination with DEC in patients with high-risk MDS and AML. Eight of sixteen (50%) patients with HR-MDS achieved CR or marrow CR. Notably, none of the responding patients had disease recurrence, with treatment durations ranging from 3.4 to 18.6 months [84]. The manufacturer, however, ended the development of sabatolimab after its phase 3 trial (NCT04266301) failed to meet the primary endpoint (overall survival) when using sabatolimab in combination with azacitidine in the treatment of high- or very-high-risk MDS and CMML-2.

### 2.4. Adoptive Cell Therapy (ACT)

CAR T-cell therapy involves collecting a patient’s autologous T cells via leukapheresis, genetically modifying them to express a chimeric antigen receptor (CAR) that targets a specific antigen, and then reintroducing the cells into the patient. Unlike in the case of lymphoid malignancies, none of the ACT products for AML have progressed to advanced stages of development. The reasons for this disparity are unique to AML. The time required for cell production, typically ranging from 3 to 5 weeks, is excessively long in the case of AML, necessitating bridging therapies and often introducing additional toxicities prior to the administration of CAR-T cells. In addition, the high genetic and molecular clonal heterogeneity of antigens in myeloid malignancies means that there is no ideal surface antigen target. Additionally, targeting antigens such as CD123 and CD33 that are shared by leukemic cells and hematopoietic stem cells makes on-target off-leukemia toxicities a significant limiting aspect of treatment, reducing the efficacy of cell therapy while magnifying the potential for systemic off-tumor toxicities. Presently, most of the ongoing clinical trials of CAR-T cells in AML target lineage-specific antigens such as CD33 and CD123 [85].

Shah et al. reported the findings of a phase 1/2 trial of CD33 CAR T-cells for children and young adults with R/R AML. Out of the 24 subjects who were enrolled, 50% of whom had a prior HSCT, 2 achieved CR [86]. In a phase I trial of anti-CD33 CAR T-cells with a 4-1BB co-stimulatory domain, ten patients with R/R AML were enrolled and only three ultimately received the cells. Unfortunately, none of these patients demonstrated a response to treatment, while two experienced CRS and one experienced ICANS [87].

Budde et al. reported more promising results of a phase I study of an anti-CD123 CAR that contained an anti-CD123 single-chain variable fragment with a CD28 co-stimulatory domain. All six patients in the AML cohort had refractory AML following allo-HSCT, with a median of four prior lines of therapy. One patient achieved MLFS that lasted for two months and then received a second infusion three months later with blast reduction from 77.9% to 0.9% after 35 days. One patient achieved CR and proceeded to a second allo-HSCT with subsequent restaging on day +161 showing MRD-negative CR. A third patient who achieved CR prior to treatment remained in CR at day 28 and then proceeded to a second allo-HSCT. Five patients experienced cytokine release syndrome (CRS) with a maximum grade of 2 [88].

C-type lectin-like molecule 1 (CLL-1) is an AML-associated antigen that has garnered researchers’ attention because it is preferentially expressed on AML stem cells and blasts while being absent on normal hematopoietic stem cells [89]. In a phase1/2 trial of a pediatric cohort with R/R AML, four out of eight patients achieved MLFS and MRD negativity, while one patient achieved MRD-positive MLFS. Additionally, one patient achieved CRi, one patient achieved partial response (PR), and one patient had stable disease (SD) but demonstrated the clearance of CLL-1 positive AML blasts. All patients developed CRS, with the maximum severity being grade 2 [90].

CD123 is another CAR T-cell antigen being explored, and early clinical trials of CD123-targeting CAR demonstrated antileukemic activity in AML cell lines [91,92], as well as an MDS xenograft model [93]. Xie et al. found in preclinical models that T-cells co-expressing bicistronic CD123 and CLL-1 CARs are effective in eradicating AML blasts [94]. Martinez et al. explored developing a non-MHC/HLA restricted healthy donor CAR T-cell line targeting CD123 in preclinical models, the premise for which was that T-cells donated from healthy donors would be more robust in their “fitness” and provide an effective “off-the-shelf” CAR T-cell option. This CAR, with a 4-1BB co-stimulatory domain, showed cytotoxicity against CD123 AML cell lines. Importantly, when mice who had cleared the initial AML graft were rechallenged with tumor cells after 40 days, while in the presence of daily IL-15 administration, CAR T-cells demonstrated ongoing persistence and cytotoxicity [95].

The FLT3 receptor promotes cell differentiation and the proliferation of hematopoietic stem cells. Activating ITD mutations in *FLT3* are present in up to 30% of patients with AML [96]. FLT3-ITD is associated with a poor prognosis in AML, and FLT3- inhibitors are now part of the standard treatment of FLT3-positive AML [96]. While preclinical models have demonstrated promising activity of anti-FLT3 CAR T-cells [97,98,99], trials have yet to demonstrate efficacy in human subjects. Presently, a phase 1/2 study is recruiting patients to evaluate the safety and efficacy of anti-FLT3 CAR T-cells in the treatment of FLT3-positive R/R AML (NCT05023707).

A novel approach to utilizing CAR T-cells is the use of CD7 CAR T-cells. CD7 is expressed by the leukemic blasts and malignant progenitor cells of approximately 30% of AML patients, but is absent on normal myeloid and erythroid cells [100]. Hu et al. reported the sequential use of CD7 CAR T-cell therapy and haploidentical allo-HSCT without myeloablation or pharmacologic GVHD prophylaxis in CD7-positive leukemias or lymphomas (seven out of the ten treated patients had AML). After CAR T-cell therapy, all ten patients had complete remission with incomplete hematologic recovery. With a median follow up of 15.1 months after CAR T-cell therapy, six patients remained in MRD-negative CR, two experienced a relapse of CD7-negative leukemia, and one patient died of septic shock at 3.7 months. Although with a reported serious adverse outcome, this approach still holds the promise of being a potential treatment option for patients otherwise ineligible for conventional HSCT [101].

Another strategy in adoptive cellular therapy is the engineering of T cells to express T-cell receptors (TCR) targeting the hematopoietic-specific minor histocompatibility antigen HA-1. This strategy is being investigated to treat the recurrence of myeloid neoplasms after allo-HSCT. Data from a phase 1 trial suggests that HA-1 TCT therapy appears to be safe and shows signs of clinical efficacy [102].

### 2.5. Allogeneic Stem Cell Transplantation

Allo-HSCT remains the only potentially curative option for fit adults with adverse-risk AML and HR-MDS, representing the ultimate immune therapy effect [100]. The survival benefit is offset by transplant-related mortality, necessitating the accurate assessment of patients who would most likely benefit from this treatment. The European Leukemia Network (ELN 2022) prognostic system stratifies AML into favorable, intermediate, and adverse risk categories [103]. Allo-HSCT is the consolidation therapy of choice for patients in CR1 with an estimated relapse risk exceeding 35% to 40%, including those with adverse-risk AML or non-adverse-risk disease with MRD persistence [104].

Prognostic systems have also been developed for MDS to identify patients at the highest risk of progressing to AML and having poorer survival outcomes. The International Prognostic Scoring System (IPSS) [4] and revised-IPSS prognostic scoring system [5] stratify patients into low, intermediate, and high risk groups based on their cytogenetic risk, percentage of marrow blasts, and number of cytopenias. With the emergence of next-generation sequencing, the molecular IPSS (IPSS-M) incorporated mutations with prognostic implications, further improving the prognostication power of IPSS and IPSS-M [7,105,106]. Patients with IPSS-R > 3.5 or IPSS-M > 0 (moderate high, high, and very high) should be considered for allo-HSCT [107,108]. Barriers to allo-HSCT include patients’ advanced age and multiple comorbidities, which are common in this typically older patient population.

### 2.6. DLI

Donor lymphocyte infusion (DLI) is a treatment modality that infuses peripheral blood lymphocytes from the original allo-HSCT donor into the recipient, thereby inducing a graft-versus-leukemia (GVL) effect [109]. This, however, comes with an increased risk of graft-versus-host disease (GVHD). The frequency of GVHD depends on a number of factors, including the total cell number infused [110] and cell manipulation, with CD8+-depleted DLI showing a lower rate of severe acute GVHD [111]. Host lymphodepletion prior to DLI is also associated with more severe GVHD [112].

DLI can achieve durable remissions in patients who relapse after allo-HSCT [113]. The first successful demonstration of the benefit of DLI for the treatment of relapsed disease was reported in three patients with relapsed chronic myelogenous leukemia (CML) after allo-HSCT, for whom treatment with DLI resulted in complete hematologic and molecular remissions [114]. Among patients with relapsed CML who received DLI, those in the chronic phase had higher rates of remission compared to those in the accelerated/blastic phase, with remission rates of 75% for those in chronic phase versus 12.5 to 33% of patients in the accelerated/blastic phase [115].

DLI, however, is less effective in MDS and AML. Schmid et al. published data on 399 patients who received allo-HSCT, 171 of whom received DLI for relapse after allo-HSCT. In total, 34% of the patients achieved remission after DLI while 66% showed persistent disease. Survival among DLI recipients is predicted by several factors, including a lower tumor burden at relapse (<35% of bone marrow blasts; *p* = 0.006), favorable cytogenetics (*p* = 0.004), remission status at the time of DLI (*p* < 0.0001), and female gender (*p* = 0.02) [116].

Minculescu et al. presented findings from a single-center study of patients with AML (n = 38) and MDS (n = 12) who received DLI following relapse post-allo-HSCT. The OS rates were 59% at 2 years and 20% at 5 years. Of note, the patients in this analysis had a relatively long time from transplant to relapse (median 17 months), indicating a cohort with potentially less aggressive disease. In this study, achieving CR prior to DLI did not demonstrate a significant impact on survival outcomes [117].

Pre-emptive DLI for patients with AML and MDS who have mixed chimerism or molecular/cytogenetic relapse after allo-HSCT can reduce relapse and improve survival outcomes. Krishnamurthy et al. evaluated the efficacy of pre-emptive and therapeutic DLI (after disease recurrence) in 113 patients with MDS or AML after allo-HSCT. The recipients of pre-emptive DLI (n = 62) had an estimated 5-year OS of 80% and an event-free survival of 65%. Those who received therapeutic DLI (n = 51) had an estimated 5-year OS of 40% and a 5-year relapse/progression rate of 69% [118]. Schmid reported the results of a registry-based analysis that evaluated the efficacy of prophylactic DLI in acute leukemia in 89 matched pairs. In patients with high-risk AML, those who received DLI and were followed for a median of 9.2 years had a 5-year OS of 69.8%, compared to 40.2% among controls who were followed for a median duration of 7.3 years (*p* = 0.027) [119].

Minor histocompatibility antigens (mHAs) are highly immunogenic antigens that contribute to polymorphisms between the allo-HSCT recipient and donor [120]. Harnessing T-cell responses to mHAs restricted to hematopoietic tissue is one means of mediating a GVL effect without instigating GVHD [121]. The early post allo-HSCT infusion of T cells engineered to target such mHAs can potentially eliminate residual leukemic cells, particularly when the mHAs are mismatched between allo-HSCT recipient and donor. In a phase 1 study, Malki et al. studied the preliminary efficacy of TSC-100 and TSC-101, allogeneic donor-derived T-cell receptor-engineered T (TCR-T) cells that target mHAs HA-1 and HA-2, respectively, in adult patients with AML, MDS, and ALL who received haploidentical HSCT. Upon count recovery after allo-HSCT, patients in treatment arms received a single dose of TSC-100 or TSC-101 at dose level 1 or repeated doses at dose levels 2 and 3. Five patients were enrolled in the treatment arm and three in the control arm. TSC-100/101 expansion and activation were observed 7–14 days after dosing, with ongoing persistence at a longest follow-up of 138 days. Two of the treatment patients who were MRD-positive pre-HSCT became MRD-negative after HSCT and subsequent TSC-100/101 treatment [122]. The use of engineered T cells targeting mHAs in hematopoietic cells is a potential strategy to allow patients to reach an MRD-negative status prior to HSCT or be utilized as a means to augment the GVL effect of hematopoietic grafts in patients who receive allo-HSCT.

### 2.7. Vaccines

Vaccines in myeloid malignancies aim to induce cellular and humoral immune responses that recognize cancer antigens specific to myeloid blasts. In order to be effective, the tumor-associated antigens (TAAs) need to be immunogenic, highly expressed, and unique to myeloid blasts. Among the antigens being investigated in leukemogenesis are Wilms’ tumor 1 (*WT1*) [123], proteinase 3 [124], and mucin 1 protein [125].

WT1 is a transcription factor involved in the transcription of growth factors and pro-oncogenic genes such as c-myc and bcl-2 [126]. Brayer et al. assessed the safety and immunogenicity of a polyvalent WT1 peptide vaccine delivered to patients with WT1-positive AML or HR-MDS after at least one prior line of therapy. The vaccination regimen consisted of six injections administered biweekly, followed by monthly injections until patients either completed twelve vaccinations or experienced disease relapse or progression. Immune responses were evaluated by delayed-type hypersensitivity testing and T-cell IFNγ ELISPOT at specified intervals. Two of fourteen patients with AML, both of whom were in CR2, demonstrated relapse-free survival exceeding 1 year. One of two patients with high-risk MDS experienced a sustained reduction in transfusion dependence [127]. Di Stasi et al. reported the findings of a systematic review of data from nine clinical trials investigating WT1 peptide vaccination in patients diagnosed with MDS or AML. A statistically significant correlation was observed between the induction of WT1-specific T cells and reduction in WT1 mRNA levels. Out of the 51 patients eligible for analysis, 25 demonstrated clinical, morphological, or molecular responses, with 3 patients achieving and maintaining remission for more than 8 years from the time of vaccination [128].

A vaccine combining WT1 with PR1 was investigated by Rezvani et al. PR1 is an HLA-A*0201- restricted peptide derived from neutrophil elastase and proteinase-3 and is highly expressed in myeloid malignancies [129]. Eight patients with myeloid malignancies, including AML in CR, CML in its chronic phase, and MDS, were enrolled in this phase 1 study. CD8+ T-cells targeting PR1 or WT1 were detected in eight of eight patients after a single vaccination and this was associated with a decrease in *WT1* mRNA expression [130].

The receptor for hyaluronic acid mediated motility (RHAMM) is an immunogenic antigen that is strongly expressed in hematologic malignancies [131]. Greiner et al. reported the results of a phase 1/2 study that enrolled nine patients with AML, MDS, and multiple myeloma. Four out of nine patients demonstrated a positive immunologic response, including an increase in RHAMM-specific CD8+ T-cells. Three patients demonstrated evidence of a clinical effect. Among them, two patients with MDS showed notable improvements: one experienced a reduction in leukemic blasts in the bone marrow, and the second had improvement in cytopenias. One patient with multiple myeloma had a reduction in serum-free light chains [132].

Treatment with HMA in MDS results in the upregulation of previously methylated and silenced tumor genes that express antigens such as NY-ESO-1, MAGE-A3, PRAME, and WT1 [133]. A phase 1 trial administered a peptide vaccine to patients with MDS who demonstrated a treatment response to six courses of azacitidine monotherapy. The vaccine targets were NY-ESO-1, MAGE-A3, PRAME, and WT1 antigens. Five patients were enrolled and received the vaccine, all of whom progressed to AML with a mean time to progression of 4.9 months and survival of 17 months after the initial MDS diagnosis. The trial was terminated early due to the absence of evidence of clinical benefits [134]. Overall, vaccines targeting leukemogenic antigens, while showing a promising immunologic response, have yet to demonstrate sustained clinical benefits in large patient cohorts [135,136,137,138]. Other vaccine strategies being explored are those that utilize transplant donor-derived dendritic cells, as well as vaccines used in combination with CAR T-cells [73,139].

### 2.8. NK Cells

Natural killer (NKs) cells are the effector lymphocytes of the innate immune system. NK cells eliminate target cells via the production of perforin and granzyme B, a direct cellular killing mechanism. Additionally, they utilize the indirect means of ADCC. NK cells play a critical role in cancer immune surveillance [140,141] and various strategies have been proposed to incorporate NK cells in relapse prevention after allo-HSCT.

Bednarski et al. treated nine pediatric and young adult patients with memory-like NK cells following a relapse of AML post-HSCT. NK cells from the original stem cell donors were stimulated ex vivo with interleukins 12, 15, and 18 to expand a memory-like NK cell population that had enhanced antileukemia responses. Subsequent to NK cell infusion, four out of eight evaluable patients achieved CR by day 28, two patients remained in remission for over three months, and one patient remained in remission for more than 2 years [142]. This study demonstrated a proof of concept for utilizing NK cells for their antileukemic effects.

Mansour et al. engineered an allogeneic human NK cell line with a CAR-recognizing FLT3 and designed these cells to secrete soluble interleukin-15 to enhance in vivo NK cell persistence. These allogeneic FLT3 CAR_sIL-15 NK cells significantly prolonged the survival of an orthotopic patient-derived xenograft AML model when compared with control NK cells. Importantly, the FLT3 CAR_sIL-15 NK cells demonstrated no cytotoxicity against healthy blood mononuclear cells or hematopoietic stem cells [143].

Bajel et al. reported the results of a phase 1/2 trial of SAR’579, a trifunctional anti-CD123 NKp46xCD16 engager that facilitates the formation of a cytolytic synapse between NK-cells and CD123-positive R/R AML, B-cell acute lymphoblastic leukemia, or HR-MDS tumor cells. SAR’579 was administered intravenously for three 28-day induction cycles. The study enrolled 42 patients with R/R AML and one patient with HR-MDS. Patients received a median of two prior lines of therapy, with 13 patients having undergone prior allo-HSCT. The combined CR/CRi rate was 12% (5/42 with R/R AML). Grade ≥3 adverse events were reported in 28 pts (65.1%), but none resulted in the permanent discontinuation of SAR’579, and no dose-limiting toxicities were observed up to the highest dose of 6000 µg/kg once weekly [144].

## 3. Future Directions

Immunotherapy, in the form of allo-HSCT, has long been the only route for a cure in MDS and AML. In the case of MDS, attempts at further harnessing the strength of the immune system to target clones came in the form of immune checkpoint inhibitors (PD-L1, CTLA-4, and CD47), but failed to show improvement over HMAs. Therapeutic vaccines have also been explored, but robust immunologic responses have not been consistently demonstrated. Effective immune therapy requires a healthy cytotoxic T-cell response, along with optimally functioning innate immunity and antigen-presenting cells. Combination strategies, such as vaccines with ICIs, CAR-T cells or antibodies, need to be considered in large clinical trials. Using these modalities after allo-HSCT in order to harness the donor immune system might prove more effective, although it may increase the risk of GVHD. Genetically altering the immunomodulatory effects of mesenchymal stem cells (MSCs) in order to increase the expression of interleukin-12 and interleukin-17 can help to alter the “inflamed” tumor microenvironment and also help activate CAR T-cells against tumor cells [134].

## 4. Conclusions

Immunotherapy for myeloid malignancies is a developing field. While allo-HSCT remains the only potentially curative treatment option for these conditions, significant progress is being made in identifying targetable antigens within these heterogenous diseases. Considerable advancements are still necessary in order to understand the immunologic profiles of AML and MDS as well as to determine the optimal immunotherapeutic strategies necessary to achieve durable remissions for patients with these disorders.

## References

[1] Sekeres M.A., Taylor J. (2022). Diagnosis and Treatment of Myelodysplastic Syndromes: A Review. JAMA.

[2] Niederwieser C., Kroger N. (2024). Hematopoietic cell transplantation (HCT) in MDS patients of older age. Leuk Lymphoma.

[3] Jentzsch M., Bischof L., Ussmann J., Backhaus D., Brauer D., Metzeler K.H., Schwind S. (2022). Prognostic Impact of the 2022 European Leukemia Net Risk Classification in Patients with Acute Myeloid Leukemia Undergoing Allogeneic Stem Cell Transplantation. Blood.

[4] Greenberg P., Cox C., LeBeau M.M., Fenaux P., Morel P., Sanz G., Sanz M., Vallespi T., Hamblin T., Oscier D. (1997). International scoring system for evaluating prognosis in myelodysplastic syndromes. Blood.

[5] Greenberg P.L., Tuechler H., Schanz J., Sanz G., Garcia-Manero G., Solé F., Bennett J.M., Bowen D., Fenaux P., Dreyfus F. (2012). Revised International Prognostic Scoring System for Myelodysplastic Syndromes. Blood.

[6] Zeidan A.M., Platzbecker U., Bewersdorf J.P., Stahl M., Adès L., Borate U., Bowen D.T., Buckstein R.J., Brunner A.M., E Carraway H. (2023). Consensus proposal for revised International Working Group response criteria for higher risk myelodysplastic syndromes. Blood.

[7] Bernard E., Tuechler H., Greenberg P.L., Hasserjian R.P., Ossa J.E.A., Nannya Y., Devlin S.M., Creignou M., Pinel P., Monnier L. (2022). Molecular International Prognostic Scoring System for Myelodysplastic Syndromes. NEJM Évid..

[8] Arber D.A., Orazi A., Hasserjian R.P., Borowitz M.J., Calvo K.R., Kvasnicka H.-M., Wang S.A., Bagg A., Barbui T., Branford S. (2022). International Consensus Classification of Myeloid Neoplasms and Acute Leukemias: Integrating morphologic, clinical, and genomic data. Blood.

[9] Awada H., Gurnari C., Xie Z., Bewersdorf J.P., Zeidan A.M. (2023). What’s Next after Hypomethylating Agents Failure in Myeloid Neoplasms? A Rational Approach. Cancers.

[10] Santini V. (2019). How I treat MDS after hypomethylating agent failure. Blood.

[11] Cherry E.M., Abbott D., Amaya M., McMahon C., Schwartz M., Rosser J., Sato A., Schowinsky J.T., Inguva A., Minhajuddin M. (2021). Venetoclax and azacitidine compared with induction chemotherapy for newly diagnosed patients with acute myeloid leukemia. Blood Adv..

[12] Vago L., Gojo I. (2020). Immune escape and immunotherapy of acute myeloid leukemia. J. Clin. Investig..

[13] Zhou W., Yu J., Li Y., Wang K. (2022). Neoantigen-specific TCR-T cell-based immunotherapy for acute myeloid leukemia. Exp. Hematol. Oncol..

[14] Roerden M., Nelde A., Walz J.S. (2019). Neoantigens in Hematological Malignancies-Ultimate Targets for Immunotherapy?. Front. Immunol..

[15] Daver N., Alotaibi A.S., Bucklein V., Subklewe M. (2021). T-cell-based immunotherapy of acute myeloid leukemia: Current concepts and future developments. Leukemia.

[16] Lichtenegger F.S., Krupka C., Haubner S., Kohnke T., Subklewe M. (2017). Recent developments in immunotherapy of acute myeloid leukemia. J. Hematol. Oncol..

[17] Kirkey D.C., Loeb A.M., Castro S., McKay C.N., Perkins L., Pardo L., Leonti A.R., Tang T.T., Loken M.R., Brodersen L.E. (2023). Therapeutic targeting of PRAME with mTCRCAR T cells in acute myeloid leukemia. Blood Adv..

[18] Schorr C., Perna F. (2022). Targets for chimeric antigen receptor T-cell therapy of acute myeloid leukemia. Front. Immunol..

[19] de Visser K.E., Joyce J.A. (2023). The evolving tumor microenvironment: From cancer initiation to metastatic outgrowth. Cancer Cell.

[20] Dysthe M., Parihar R. (2020). Myeloid-Derived Suppressor Cells in the Tumor Microenvironment. Adv. Exp. Med. Biol..

[21] Mantovani A., Allavena P., Marchesi F., Garlanda C. (2022). Macrophages as tools and targets in cancer therapy. Nat. Rev. Drug Discov..

[22] Ephraim R., Fraser S., Nurgali K., Apostolopoulos V. (2022). Checkpoint Markers and Tumor Microenvironment: What Do We Know?. Cancers.

[23] Kim S.K., Cho S.W. (2022). The Evasion Mechanisms of Cancer Immunity and Drug Intervention in the Tumor Microenvironment. Front. Pharmacol..

[24] Fathi E., Farahzadi R., Sheervalilou R., Sanaat Z., Vietor I. (2020). A general view of CD33(+) leukemic stem cells and CAR-T cells as interesting targets in acute myeloblatsic leukemia therapy. Blood Res..

[25] Liu J., Tong J., Yang H. (2022). Targeting CD33 for acute myeloid leukemia therapy. BMC Cancer.

[26] Feldman E.J., Brandwein J., Stone R., Kalaycio M., Moore J., O’Connor J., Wedel N., Roboz G.J., Miller C., Chopra R. (2005). Phase III randomized multicenter study of a humanized anti-CD33 monoclonal antibody, lintuzumab, in combination with chemotherapy, versus chemotherapy alone in patients with refractory or first-relapsed acute myeloid leukemia. J. Clin. Oncol..

[27] Petersdorf S.H., Kopecky K.J., Slovak M., Willman C., Nevill T., Brandwein J., Larson R.A., Erba H.P., Stiff P.J., Stuart R.K. (2013). A phase 3 study of gemtuzumab ozogamicin during induction and postconsolidation therapy in younger patients with acute myeloid leukemia. Blood.

[28] Burnett A.K., Hills R.K., Milligan D., Kjeldsen L., Kell J., Russell N.H., Yin J.A., Hunter A., Goldstone A.H., Wheatley K. (2011). Identification of Patients with Acute Myeloblastic Leukemia Who Benefit from the Addition of Gemtuzumab Ozogamicin: Results of the MRC AML15 Trial. J. Clin. Oncol..

[29] Freeman S.D., Thomas A., Thomas I., Hills R.K., Vyas P., Gilkes A., Metzner M., Jakobsen N.A., Kennedy A., Moore R. (2023). Fractionated vs single-dose gemtuzumab ozogamicin with determinants of benefit in older patients with AML: The UK NCRI AML18 trial. Blood.

[30] Hills R.K., Castaigne S., Appelbaum F.R., Delaunay J., Petersdorf S., Othus M., Estey E.H., Dombret H., Chevret S., Ifrah N. (2014). Addition of gemtuzumab ozogamicin to induction chemotherapy in adult patients with acute myeloid leukaemia: A meta-analysis of individual patient data from randomised controlled trials. Lancet Oncol..

[31] Castaigne S., Pautas C., Terré C., Raffoux E., Bordessoule D., Bastie J.-N., Legrand O., Thomas X., Turlure P., Reman O. (2012). Effect of gemtuzumab ozogamicin on survival of adult patients with de-novo acute myeloid leukaemia (ALFA-0701): A randomised, open-label, phase 3 study. Lancet.

[32] Norsworthy K.J., Ko C.W., Lee J.E., Liu J., John C.S., Przepiorka D. (2018). FDA Approval Summary: Mylotarg for Treatment of Patients with Relapsed or Refractory CD33-Positive Acute Myeloid Leukemia. Oncologist.

[33] Gbadamosi M., Meshinchi S., Lamba J.K. (2018). Gemtuzumab ozogamicin for treatment of newly diagnosed CD33-positive acute myeloid leukemia. Future Oncol..

[34] Ravandi F., Estey E., Jones D., Faderl S., O’Brien S., Fiorentino J., Pierce S., Blamble D., Estrov Z., Wierda W. (2009). Effective treatment of acute promyelocytic leukemia with all-trans-retinoic acid, arsenic trioxide, and gemtuzumab ozogamicin. J. Clin. Oncol..

[35] Abedin S.M., Murthy G.S.G., Desai A., Chen M., Atallah E.L. (2023). Sequential salvage chemotherapy and lintuzumab-Ac225 results in deep responses and prolonged survival in adverse risk relapsed/refractory AML and in AML patients that received prior venetoclax therapy. J. Clin. Oncol..

[36] El Achi H., Dupont E., Paul S., Khoury J.D. (2020). CD123 as a Biomarker in Hematolymphoid Malignancies: Principles of Detection and Targeted Therapies. Cancers.

[37] E Hogge D., Yalcintepe L., Wong S.-H., Gerhard B., E Frankel A. (2006). Variant diphtheria toxin-interleukin-3 fusion proteins with increased receptor affinity have enhanced cytotoxicity against acute myeloid leukemia progenitors. Clin. Cancer Res..

[38] Pemmaraju N., Konopleva M. (2020). Approval of tagraxofusp-erzs for blastic plasmacytoid dendritic cell neoplasm. Blood Adv..

[39] Mani R., Goswami S., Gopalakrishnan B., Ramaswamy R., Wasmuth R., Tran M., Mo X.K., Gordon A., Gordon D., Lucas D.M. (2018). The interleukin-3 receptor CD123 targeted SL-401 mediates potent cytotoxic activity against CD34(+)CD123(+) cells from acute myeloid leukemia/myelodysplastic syndrome patients and healthy donors. Haematologica.

[40] Lane A.A., Stein A.S., Garcia J.S., Garzon J.L., Galinsky I., Luskin M.R., Stone R.M., Winer E.S., Leonard R., Mughal T.I. (2021). Safety and Efficacy of Combining Tagraxofusp (SL-401) with Azacitidine or Azacitidine and Venetoclax in a Phase 1b Study for CD123 Positive AML, MDS, or BPDCN. Blood.

[41] Minetto P., Rosellini S., Guolo F., Tedone E., Audisio E., Cattaneo C., Bocchia M., Fracchiolla N., Martelli M.P., Crea E. (2023). Single Agent Tagraxofusp in Relapsed/Refractory CD123-Positive Acute Myeloid Leukemia: A Preliminary Analysis of Italian Gimema AML2020 Trial. Blood.

[42] Mouhayar E.N., Hammond D., Lopez-Mattei J., Banchs J., Konopleva M., Pemmaraju N. (2021). Reversible Myocardial Edema Secondary to Tagraxofusp-Induced Capillary Leak Syndrome. JACC Cardio Oncol..

[43] Daver N.G., Montesinos P., DeAngelo D.J., Wang E.S., Papadantonakis N., Todisco E., Sweet K.L., Pemmaraju N., Lane A.A., Torres-Miñana L. (2024). Pivekimab sunirine (IMGN632), a novel CD123-targeting antibody-drug conjugate, in relapsed or refractory acute myeloid leukaemia: A phase 1/2 study. Lancet Oncol..

[44] Kuruvilla V.M., Zhang Q., Daver N., Watkins K., Sloss C.M., Zweidler-McKay P.A., Romanelli A., Konopleva M. (2020). Combining IMGN632, a Novel CD123-Targeting Antibody Drug Conjugate with Azacitidine and Venetoclax Facilitates Apoptosis In Vitro and Prolongs Survival In Vivo in AML Models. Blood.

[45] Daver N., Montesinos P., Aribi A., Altman J.K., Wang E.S., Roboz G.J., Burke P.W., Gaidano G., Walter R.B., Thomas X. (2022). Broad Activity for the Pivekimab Sunirine (PVEK, IMGN632), Azacitidine, and Venetoclax Triplet in High-Risk Patients with Relapsed/Refractory Acute Myeloid Leukemia (AML). Blood.

[46] Tapia-Galisteo A., Alvarez-Vallina L., Sanz L. (2023). Bi- and trispecific immune cell engagers for immunotherapy of hematological malignancies. J. Hematol. Oncol..

[47] Smits N.C., Sentman C.L. (2016). Bispecific T-Cell Engagers (BiTEs) as Treatment of B-Cell Lymphoma. J. Clin. Oncol..

[48] Przepiorka D., Ko C.W., Deisseroth A., Yancey C.L., Candau-Chacon R., Chiu H.J., Gehrke B.J., Gomez-Broughton C., Kane R.C., Kirshner S. (2015). FDA Approval: Blinatumomab. Clin. Cancer Res..

[49] Kontermann R.E. (2011). Strategies for extended serum half-life of protein therapeutics. Curr. Opin. Biotechnol..

[50] Gogesch P., Dudek S., van Zandbergen G., Waibler Z., Anzaghe M. (2021). The Role of Fc Receptors on the Effectiveness of Therapeutic Monoclonal Antibodies. Int. J. Mol. Sci..

[51] Balendran S., Tam C., Ku M. (2023). T-Cell Engaging Antibodies in Diffuse Large B Cell Lymphoma—An Update. J. Clin. Med..

[52] Krupka C., Kufer P., Kischel R., Zugmaier G., Bögeholz J., Köhnke T., Lichtenegger F.S., Schneider S., Metzeler K., Fiegl M. (2014). CD33 target validation and sustained depletion of AML blasts in long-term cultures by the bispecific T-cell–engaging antibody AMG 330. Blood.

[53] Sidori A., Cerchione C., Daver N., DiNardo C., Garcia-Manero G., Konopleva M., Jabbour E., Ravandi F., Kadia T., Burguera A.d.l.F. (2021). Immunotherapy in Acute Myeloid Leukemia: Where We Stand. Front. Oncol..

[54] Westervelt P., Cortes J.E., Altman J.K., Long M., Oehler V.G., Gojo I., Guenot J., Chun P., Roboz G.J. (2019). Phase 1 First-in-Human Trial of AMV564, a Bivalent Bispecific (2:2) CD33/CD3 T-Cell Engager, in Patients with Relapsed/Refractory Acute Myeloid Leukemia (AML). Blood.

[55] Ravandi F., Bashey A., Foran J., Stock W., Mawad R., Short N., Yilmaz M., Kantarjian H., Odenike O., Patel A. (2023). Phase 1 study of vibecotamab identifies an optimized dose for treatment of relapsed/refractory acute myeloid leukemia. Blood Adv..

[56] Uy G.L., Aldoss I., Foster M.C., Sayre P.H., Wieduwilt M.J., Advani A.S., Godwin J.E., Arellano M.L., Sweet K.L., Emadi A. (2021). Flotetuzumab as salvage immunotherapy for refractory acute myeloid leukemia. Blood.

[57] Riva C., Vernarecci C., Minetto P., Goda R., Greppi M., Pesce S., Chies M., Zecchetti G., Ferro B., Maio E. (2023). Harnessing Immune Response in Acute Myeloid Leukemia. J. Clin. Med..

[58] Braciak T.A., Roskopf C.C., Wildenhain S., Fenn N.C., Schiller C.B., Schubert I.A., Jacob U., Honegger A., Krupka C., Subklewe M. (2018). Dual-targeting triplebody 33-16-123 (SPM-2) mediates effective redirected lysis of primary blasts from patients with a broad range of AML subtypes in combination with natural killer cells. OncoImmunology.

[59] Yang H., Bueso-Ramos C., Dinardo C., Estecio M.R., Davanlou M., Geng Q.-R., Fang Z., Nguyen M., Pierce S., Wei Y. (2014). Expression of PD-L1, PD-L2, PD-1 and CTLA4 in myelodysplastic syndromes is enhanced by treatment with hypomethylating agents. Leukemia.

[60] Stahl M., Goldberg A.D. (2019). Immune Checkpoint Inhibitors in Acute Myeloid Leukemia: Novel Combinations and Therapeutic Targets. Curr. Oncol. Rep..

[61] Perna F., Espinoza-Gutarra M.R., Bombaci G., Farag S.S., Schwartz J.E. (2022). Immune-Based Therapeutic Interventions for Acute Myeloid Leukemia. Cancer Treat Res..

[62] Chan T., Yarchoan M., Jaffee E., Swanton C., Quezada S., Stenzinger A., Peters S. (2019). Development of tumor mutation burden as an immunotherapy biomarker: Utility for the oncology clinic. Ann. Oncol..

[63] Salik B., Smyth M.J., Nakamura K. (2020). Targeting immune checkpoints in hematological malignancies. J. Hematol. Oncol..

[64] Yang X., Ma L., Zhang X., Huang L., Wei J. (2022). Targeting PD-1/PD-L1 pathway in myelodysplastic syndromes and acute myeloid leukemia. Exp. Hematol. Oncol..

[65] Chao M.P., Takimoto C.H., Feng D.D., McKenna K., Gip P., Liu J., Volkmer J.-P., Weissman I.L., Majeti R., Gibb P. (2019). Therapeutic Targeting of the Macrophage Immune Checkpoint CD47 in Myeloid Malignancies. Front. Oncol..

[66] Kikushige Y. (2022). Clinical roles of TIM-3 in myeloid malignancies and its importance in cellular therapy. Blood Cell Ther. Off. J. APBMT.

[67] Ricciuti B., Wang X., Alessi J.V., Rizvi H., Mahadevan N.R., Li Y.Y., Polio A., Lindsay J., Umeton R., Sinha R. (2022). Association of High Tumor Mutation Burden in Non–Small Cell Lung Cancers with Increased Immune Infiltration and Improved Clinical Outcomes of PD-L1 Blockade Across PD-L1 Expression Levels. JAMA Oncol..

[68] Wang P., Chen Y., Wang C. (2021). Beyond Tumor Mutation Burden: Tumor Neoantigen Burden as a Biomarker for Immunotherapy and Other Types of Therapy. Front. Oncol..

[69] Daver N., Konopleva M., Maiti A., Kadia T.M., DiNardo C.D., Loghavi S., Pemmaraju N., Jabbour E., Montalban-Bravo G., Tang G.L. (2021). Phase I/II Study of Azacitidine (AZA) with Venetoclax (VEN) and Magrolimab (Magro) in Patients (pts) with Newly Diagnosed Older/Unfit or High-Risk Acute Myeloid Leukemia (AML) and Relapsed/Refractory (R/R) AML. Blood.

[70] Zeidan A.M., Boss I.W., Beach C.L., Copeland W.B., Thompson E.G., Fox B.A., Hasle V.E., Ogasawara K., Cavenagh J., Silverman L.R. (2022). A randomized phase 2 trial of azacitidine with or without durvalumab as first-line therapy for higher-risk myelodysplastic syndromes. Blood Adv..

[71] Garcia-Manero G., Sasaki K., Montalban-Bravo G., Daver N.G., Jabbour E.J., Alvarado Y., DiNardo C.D., Ravandi F., Borthakur G., Bose P. (2018). A Phase II Study of Nivolumab or Ipilimumab with or without Azacitidine for Patients with Myelodysplastic Syndrome (MDS). Blood.

[72] Willingham S.B., Volkmer J.-P., Gentles A.J., Sahoo D., Dalerba P., Mitra S.S., Wang J., Contreras-Trujillo H., Martin R., Cohen J.D. (2012). The CD47-signal regulatory protein alpha (SIRPa) interaction is a therapeutic target for human solid tumors. Proc. Natl. Acad. Sci. USA.

[73] Linder K., Lulla P. (2021). Myelodysplastic syndrome and immunotherapy novel to next in-line treatments. Hum. Vaccines Immunother..

[74] Sallman D.A., Al Malki M.M., Asch A.S., Wang E.S., Jurcic J.G., Bradley T.J., Flinn I.W., Pollyea D.A., Kambhampati S., Tanaka T.N. (2023). Magrolimab in Combination with Azacitidine in Patients with Higher-Risk Myelodysplastic Syndromes: Final Results of a Phase Ib Study. J. Clin. Oncol..

[75] A Sallman D., Asch A.S., Al Malki M.M., Lee D.J., Donnellan W.B., Marcucci G., Kambhampati S., Daver N.G., Garcia-Manero G., Komrokji R.S. (2019). The First-in-Class Anti-CD47 Antibody Magrolimab (5F9) in Combination with Azacitidine Is Effective in MDS and AML Patients: Ongoing Phase 1b Results. Blood.

[76] Garcia-Manero G. (2023). Current status of phase 3 clinical trials in high-risk myelodysplastic syndromes: Pitfalls and recommendations. Lancet Haematol..

[77] Stirling E.R., Terabe M., Wilson A.S., Kooshki M., Yamaleyeva L.M., Alexander-Miller M.A., Zhang W., Miller L.D., Triozzi P.L., Soto-Pantoja D.R. (2022). Targeting the CD47/thrombospondin-1 signaling axis regulates immune cell bioenergetics in the tumor microenvironment to potentiate antitumor immune response. J. Immunother. Cancer.

[78] Chen Q., Guo X., Ma W. (2023). Opportunities and challenges of CD47-targeted therapy in cancer immunotherapy. Oncol. Res. Featur. Preclin. Clin. Cancer Ther..

[79] Yamada-Hunter S.A., Theruvath J., McIntosh B.J., Freitas K.A., Lin F., Radosevich M.T., Leruste A., Dhingra S., Martinez-Velez N., Xu P. (2024). Engineered CD47 protects T cells for enhanced antitumour immunity. Nature.

[80] Tahk S., Vick B., Hiller B., Schmitt S., Marcinek A., Perini E.D., Leutbecher A., Leutbecher C., Leutbecher A., Tast B. (2021). SIRPalpha-alphaCD123 fusion antibodies targeting CD123 in conjunction with CD47 blockade enhance the clearance of AML-initiating cells. J. Hematol. Oncol..

[81] Das M., Zhu C., Kuchroo V.K. (2017). Tim-3 and its role in regulating anti-tumor immunity. Immunol. Rev..

[82] Tan J., Tan H., Li Y. (2023). Targeting TIM-3 for hematological malignancy: Latest updates from the 2022 ASH annual meeting. Exp. Hematol. Oncol..

[83] Xu S., Zhang N., Rinne M.L., Sun H., Stein A.M. (2023). Sabatolimab (MBG453) model-informed drug development for dose selection in patients with myelodysplastic syndrome/acute myeloid leukemia and solid tumors. CPT Pharmacometrics Syst. Pharmacol..

[84] Borate U., Esteve J., Porkka K., Knapper S., Vey N., Scholl S., Garcia-Manero G., Wermke M., Janssen J., Traer E. (2019). Phase Ib Study of the Anti-TIM-3 Antibody MBG453 in Combination with Decitabine in Patients with High-Risk Myelodysplastic Syndrome (MDS) and Acute Myeloid Leukemia (AML). Blood.

[85] Dao T., Xiong G., Mun S.S., Meyerberg J., Korontsvit T., Xiang J., Cui Z., Chang A.Y., Jarvis C., Cai W. (2024). A dual-receptor T-cell platform with Ab-TCR and costimulatory receptor achieves specificity and potency against AML. Blood.

[86] Shah N.N., Azzi J., Cooper B.W., Deol A., DiPersio J., Koura D., McClune B., Muffly L.S., Mushtaq M.U., Narayan R. (2023). Phase 1/2 Study of Donor-Derived Anti-CD33 Chimeric Antigen Receptor Expressing T Cells (VCAR33) in Patients with Relapsed or Refractory Acute Myeloid Leukemia after Allogeneic Hematopoietic Cell Transplantation. Blood.

[87] Tambaro F.P., Singh H., Jones E., Rytting M., Mahadeo K.M., Thompson P., Daver N., DiNardo C., Kadia T., Garcia-Manero G. (2021). Autologous CD33-CAR-T cells for treatment of relapsed/refractory acute myelogenous leukemia. Leukemia.

[88] Budde L., Song J.Y., Kim Y., Blanchard S., Wagner J., Stein A.S., Weng L., Del Real M., Hernandez R., Marcucci E. (2017). Remissions of Acute Myeloid Leukemia and Blastic Plasmacytoid Dendritic Cell Neoplasm Following Treatment with CD123-Specific CAR T Cells: A First-in-Human Clinical Trial. Blood.

[89] Wang J., Wang W., Chen H., Li W., Huang T., Zhang W., Ling W., Lai P., Wang Y., Geng S. (2021). C-Type Lectin-Like Molecule-1 as a Biomarker for Diagnosis and Prognosis in Acute Myeloid Leukemia: A Preliminary Study. BioMed. Res. Int..

[90] Zhang H., Bu C., Peng Z., Li G., Zhou Z., Ding W., Zheng Y., He Y., Hu Z., Pei K. (2022). Characteristics of anti-CLL1 based CAR-T therapy for children with relapsed or refractory acute myeloid leukemia: The multi-center efficacy and safety interim analysis. Leukemia.

[91] Mardiros A., Dos Santos C., McDonald T., Brown C.E., Wang X., Budde L.E., Hoffman L., Aguilar B., Chang W.-C., Bretzlaff W. (2013). T cells expressing CD123-specific chimeric antigen receptors exhibit specific cytolytic effector functions and antitumor effects against human acute myeloid leukemia. Blood.

[92] Gill S., Tasian S.K., Ruella M., Shestova O., Li Y., Porter D.L., Carroll M., Danet-Desnoyers G., Scholler J., Grupp S.A. (2014). Preclinical targeting of human acute myeloid leukemia and myeloablation using chimeric antigen receptor-modified T cells. Blood.

[93] Stevens B.M., Zhang W., Pollyea D.A., Winters A., Gutman J., Smith C., Budde E., Forman S.J., Jordan C.T., Purev E. (2019). CD123 CAR T cells for the treatment of myelodysplastic syndrome. Exp. Hematol..

[94] Xie D., Jin X., Sun R., Zhang M., Lu W., Cao X., Guo R., Zhang Y., Zhao M. (2023). Bicistronic CAR-T cells targeting CD123 and CLL1 for AML to reduce the risk of antigen escape. Transl. Oncol..

[95] Martínez D.S., Tirado N., Mensurado S., Martínez-Moreno A., Romecín P., Agüera F.G., Correia D.V., Silva-Santos B., Menéndez P. (2022). Generation and proof-of-concept for allogeneic CD123 CAR-Delta One T (DOT) cells in acute myeloid leukemia. J. Immunother. Cancer.

[96] Daver N., Venugopal S., Ravandi F. (2021). FLT3 mutated acute myeloid leukemia: 2021 treatment algorithm. Blood Cancer J..

[97] Chen L., Mao H., Zhang J., Chu J., Devine S., A Caligiuri M., Yu J. (2017). Targeting FLT3 by chimeric antigen receptor T cells for the treatment of acute myeloid leukemia. Leukemia.

[98] Karbowski C., Goldstein R., Frank B., Kim K., Li C.M., Homann O., Hensley K., Brooks B., Wang X., Yan Q. (2020). Nonclinical Safety Assessment of AMG 553, an Investigational Chimeric Antigen Receptor T-Cell Therapy for the Treatment of Acute Myeloid Leukemia. Toxicol. Sci..

[99] Jetani H., Garcia-Cadenas I., Nerreter T., Thomas S., Rydzek J., Meijide J.B., Bonig H., Herr W., Sierra J., Einsele H. (2018). CAR T-cells targeting FLT3 have potent activity against FLT3−ITD+ AML and act synergistically with the FLT3-inhibitor crenolanib. Leukemia.

[100] Cornelissen J.J., Breems D., van Putten W.L., Gratwohl A.A., Passweg J.R., Pabst T., Maertens J., Beverloo H.B., Kooy M.v.M., Wijermans P.W. (2012). Comparative Analysis of the Value of Allogeneic Hematopoietic Stem-Cell Transplantation in Acute Myeloid Leukemia with Monosomal Karyotype Versus Other Cytogenetic Risk Categories. J. Clin. Oncol..

[101] Hu Y., Zhang M., Yang T., Mo Z., Wei G., Jing R., Zhao H., Chen R., Zu C., Gu T. (2024). Sequential CD7 CAR T-Cell Therapy and Allogeneic HSCT without GVHD Prophylaxis. N. Engl. J. Med..

[102] Krakow E.F., Brault M., Summers C., Cunningham T.M., A Biernacki M., Black R.G., Woodward K.B., Vartanian N., Kanaan S.B., Yeh A.C. HA-1-targeted T cell receptor (TCR) T cell therapy for recurrent leukemia after hematopoietic stem cell transplantation. Blood.

[103] Döhner H., Wei A.H., Appelbaum F.R., Craddock C., DiNardo C.D., Dombret H., Ebert B.L., Fenaux P., Godley L.A., Hasserjian R.P. (2022). Diagnosis and management of AML in adults: 2022 recommendations from an international expert panel on behalf of the ELN. Blood.

[104] Cornelissen J.J., Blaise D. (2016). Hematopoietic stem cell transplantation for patients with AML in first complete remission. Blood.

[105] Robin M., Porcher R., Ruggeri A., Blaise D., Wolschke C., Koster L., Angelucci E., Stölzel F., Potter V., Yakoub-Agha I. (2019). HLA-Mismatched Donors in Patients with Myelodysplastic Syndrome: An EBMT Registry Analysis. Biol. Blood Marrow Transplant..

[106] Kröger N., Iacobelli S., Franke G.-N., Platzbecker U., Uddin R., Hübel K., Scheid C., Weber T., Robin M., Stelljes M. (2017). Dose-Reduced versus Standard Conditioning Followed by Allogeneic Stem-Cell Transplantation for Patients with Myelodysplastic Syndrome: A Prospective Randomized Phase III Study of the EBMT (RICMAC Trial). J. Clin. Oncol..

[107] Mina A., Greenberg P.L., Deeg H.J. (2024). How I reduce and treat posttransplant relapse of MDS. Blood.

[108] Tentori C.A., Gregorio C., Robin M., Gagelmann N., Gurnari C., Ball S., Caballero Berrocal J.C., Lanino L., D’Amico S., Spreafico M. (2024). Clinical and Genomic-Based Decision Support System to Define the Optimal Timing of Allogeneic Hematopoietic Stem-Cell Transplantation in Patients with Myelodysplastic Syndromes. J. Clin. Oncol..

[109] Maurer K., Antin J.H. (2024). The graft versus leukemia effect: Donor lymphocyte infusions and cellular therapy. Front. Immunol..

[110] Bar M., Sandmaier B.M., Inamoto Y., Bruno B., Hari P., Chauncey T., Martin P.J., Storb R., Maloney D.G., Storer B. (2013). Donor Lymphocyte Infusion for Relapsed Hematological Malignancies after Allogeneic Hematopoietic Cell Transplantation: Prognostic Relevance of the Initial CD3+ T Cell Dose. Biol. Blood Marrow Transplant..

[111] Giralt S., Hester J., Huh Y., Hirsch-Ginsberg C., Rondón G., Seong D., Lee M., Gajewski J., Van Besien K., Khouri I. (1995). CD8-depleted donor lymphocyte infusion as treatment for relapsed chronic myelogenous leukemia after allogeneic bone marrow transplantation. Blood.

[112] Miller J.S., Weisdorf D.J., Burns L.J., Slungaard A., Wagner J.E., Verneris M.R., Cooley S., Wangen R., Fautsch S.K., Nicklow R. (2007). Lymphodepletion followed by donor lymphocyte infusion (DLI) causes significantly more acute graft-versus-host disease than DLI alone. Blood.

[113] Ye Y., Yang L., Yuan X., Huang H., Luo Y. (2021). Optimization of Donor Lymphocyte Infusion for AML Relapse After Allo-HCT in the Era of New Drugs and Cell Engineering. Front. Oncol..

[114] Kolb H.J., Mittermüller J., Clemm C., Holler E., Ledderose G., Brehm G., Heim M., Wilmanns W. (1990). Donor leukocyte transfusions for treatment of recurrent chronic myelogenous leukemia in marrow transplant patients. Blood.

[115] Deol A., Lum L.G. (2010). Role of donor lymphocyte infusions in relapsed hematological malignancies after stem cell transplantation revisited. Cancer Treat. Rev..

[116] Schmid C., Labopin M., Nagler A., Bornhäuser M., Finke J., Fassas A., Volin L., Gürman G., Maertens J., Bordigoni P. (2007). Donor Lymphocyte Infusion in the Treatment of First Hematological Relapse after Allogeneic Stem-Cell Transplantation in Adults with Acute Myeloid Leukemia: A Retrospective Risk Factors Analysis and Comparison with Other Strategies by the EBMT Acute Leukemia Working Party. J. Clin. Oncol..

[117] Minculescu L., Reekie J., Petersen S.L., Kornblit B.T., Schjoedt I., Andersen N.S., Andersen L.P., Fischer-Nielsen A., Haastrup E.K., Friis L.S. (2023). Donor Lymphocyte Infusion Is a Feasible Way to Improve Survival in Patients with Acute Myeloid Leukemia and Myelodysplastic Syndromes Who Relapse after Allogeneic Stem Cell Transplantation. Acta Haematol..

[118] Krishnamurthy P., Potter V.T., Barber L.D., Kulasekararaj A.G., Lim Z.Y., Pearce R.M., de Lavallade H., Kenyon M., Ireland R.M., Marsh J.C. (2013). Outcome of Donor Lymphocyte Infusion after T Cell–depleted Allogeneic Hematopoietic Stem Cell Transplantation for Acute Myelogenous Leukemia and Myelodysplastic Syndromes. Biol. Blood Marrow Transplant..

[119] Schmid C., Labopin M., Schaap N., Veelken H., Schleuning M., Stadler M., Finke J., Hurst E., Baron F., Ringden O. (2019). Prophylactic donor lymphocyte infusion after allogeneic stem cell transplantation in acute leukaemia—A matched pair analysis by the Acute Leukaemia Working Party of EBMT. Br. J. Haematol..

[120] Akatsuka Y. (2020). TCR-Like CAR-T Cells Targeting MHC-Bound Minor Histocompatibility Antigens. Front. Immunol..

[121] Olsen K.S., Jadi O., Dexheimer S., Bortone D.S., Vensko S.P., Bennett S., Tang H., Diiorio M., Saran T., Dingfelder D. (2023). Shared graft-versus-leukemia minor histocompatibility antigens in DISCOVeRY-BMT. Blood Adv..

[122] Al Malki M.M., Keyzner A., Suh H.C., Matin A., Buonomo E., Wang Y., Abelowitz N., Murray J., Macbeath G., Barton D. (2023). Initial Results of a Phase 1 Trial of TSC-100 and TSC-101, Engineered T Cell Therapies That Target Minor Histocompatibility Antigens to Prevent Relapse after Allogeneic Hematopoietic Cell Transplantation. Blood.

[123] Kreutmair S., Pfeifer D., Waterhouse M., Takács F., Graessel L., Döhner K., Duyster J., Illert A.L., Frey A.-V., Schmitt M. (2022). First-in-human study of WT1 recombinant protein vaccination in elderly patients with AML in remission: A single-center experience. Cancer Immunol. Immunother..

[124] Scheibenbogen C., Letsch A., Thiel E., Schmittel A., Mailaender V., Baerwolf S., Nagorsen D., Keilholz U. (2002). CD8 T-cell responses to Wilms tumor gene product WT1 and proteinase 3 in patients with acute myeloid leukemia. Blood.

[125] Kuball J., de Boer K., Wagner E., Wattad M., Antunes E., Weeratna R.D., Vicari A.P., Lotz C., van Dorp S., Hol S. (2011). Pitfalls of vaccinations with WT1-, Proteinase3- and MUC1-derived peptides in combination with MontanideISA51 and CpG7909. Cancer Immunol. Immunother..

[126] Sugiyama H. (2001). Wilms’ Tumor GeneWT1: Its Oncogenic Function and Clinical Application. Int. J. Hematol..

[127] Brayer J., Lancet J.E., Powers J., List A., Balducci L., Komrokji R., Pinilla-Ibarz J. (2015). WT1 vaccination in AML and MDS: A pilot trial with synthetic analog peptides. Am. J. Hematol..

[128] Di Stasi A., Jimenez A.M., Eminagawa K., Eal-Obaidi M., Erezvani K. (2015). Review of the Results of WT1 Peptide Vaccination Strategies for Myelodysplastic Syndromes and Acute Myeloid Leukemia from Nine Different Studies. Front. Immunol..

[129] Alatrash G., Molldrem J.J., Qazilbas M.H. (2018). Targeting PR1 in myeloid leukemia. Oncotarget.

[130] Rezvani K., Yong A.S.M., Mielke S., Savani B.N., Musse L., Superata J., Jafarpour B., Boss C., Barrett A.J. (2008). Leukemia-associated antigen-specific T-cell responses following combined PR1 and WT1 peptide vaccination in patients with myeloid malignancies. Blood.

[131] Hinneh J.A., Gillis J.L., Moore N.L., Butler L.M., Centenera M.M. (2022). The role of RHAMM in cancer: Exposing novel therapeutic vulnerabilities. Front. Oncol..

[132] Greiner J., Schmitt A., Giannopoulos K., Rojewski M.T., Götz M., Funk I., Ringhoffer M., Bunjes D., Hofmann S., Ritter G. (2010). High-dose RHAMM-R3 peptide vaccination for patients with acute myeloid leukemia, myelodysplastic syndrome and multiple myeloma. Haematologica.

[133] Rausch J., Ullrich E., Kühn M.W. (2023). Epigenetic targeting to enhance acute myeloid leukemia-directed immunotherapy. Front. Immunol..

[134] Holmberg-Thydén S., Dufva I.H., Gang A.O., Breinholt M.F., Schejbel L., Andersen D.M.K., Kadivar M., Svane I.M., Grønbæk K., Hadrup S.R. (2020). Therapeutic Cancer Vaccination Targeting Shared Tumor Associated Antigens in Combination with Azacitidine for High Risk Myelodysplastic Syndrome—A Phase I Clinical Trial. Blood.

[135] Keilholz U., Letsch A., Busse A., Asemissen A.M., Bauer S., Blau I.W., Hofmann W.-K., Uharek L., Thiel E., Scheibenbogen C. (2009). A clinical and immunologic phase 2 trial of Wilms tumor gene product 1 (WT1) peptide vaccination in patients with AML and MDS. Blood.

[136] Naoe T., Saito A., Hosono N., Kasahara S., Muto H., Hatano K., Ogura M., Masunari T., Tanaka M., Usuki K. (2023). Immunoreactivity to WT1 peptide vaccine is associated with prognosis in elderly patients with acute myeloid leukemia: Follow-up study of randomized phase II trial of OCV-501, an HLA class II-binding WT1 polypeptide. Cancer Immunol. Immunother..

[137] Schmitt M., Schmitt A., Rojewski M.T., Chen J., Giannopoulos K., Fei F., Yu Y., Götz M., Heyduk M., Ritter G. (2008). RHAMM-R3 peptide vaccination in patients with acute myeloid leukemia, myelodysplastic syndrome, and multiple myeloma elicits immunologic and clinical responses. Blood.

[138] Griffiths E.A., Srivastava P., Matsuzaki J., Brumberger Z., Wang E.S., Kocent J., Miller A., Roloff G.W., Wong H.Y., Paluch B.E. (2018). NY-ESO-1 Vaccination in Combination with Decitabine Induces Antigen-Specific T-lymphocyte Responses in Patients with Myelodysplastic Syndrome. Clin. Cancer Res..

[139] Avigan D., Rosenblatt J. (2018). Vaccine therapy in hematologic malignancies. Blood.

[140] Cooley S., Parham P., Miller J.S. (2018). Strategies to activate NK cells to prevent relapse and induce remission following hematopoietic stem cell transplantation. Blood.

[141] Guzman L.G.M., Keating N., Nicholson S.E. (2020). Natural Killer Cells: Tumor Surveillance and Signaling. Cancers.

[142] Bednarski J.J., Zimmerman C., Berrien-Elliott M.M., Foltz J.A., Becker-Hapak M., Neal C.C., Foster M., Schappe T., McClain E., Pence P.P. (2022). Donor memory-like NK cells persist and induce remissions in pediatric patients with relapsed AML after transplant. Blood.

[143] Mansour A.G., Teng K.-Y., Li Z., Zhu Z., Chen H., Tian L., Ali A., Zhang J., Lu T., Ma S. (2023). Off-the-shelf CAR–engineered natural killer cells targeting FLT3 enhance killing of acute myeloid leukemia. Blood Adv..

[144] Bajel A., Garciaz S., Desai P., A Huls G., Maiti A., Jongen-Lavrencic M., Boissel N., De Botton S., de Leeuw D.C., Fleming S. (2023). First-in-Human Study of the CD123 NK Cell Engager SAR443579 in Relapsed or Refractory Acute Myeloid Leukemia, B-Cell Acute Lymphoblastic Leukemia or High Risk-Myelodysplasia: Updated Safety, Efficacy, Pharmacokinetics and Pharmacodynamics. Blood.

